# Sleep Quality, Psychological Resilience, and Attachment Dimensions in Women with Unexplained and Male Factor Infertility: A Cross-Sectional Study

**DOI:** 10.3390/jcm15186953

**Published:** 2026-09-08

**Authors:** Fatma Tülücü Kalkan, İbrahim Taşkum, Fatma Sucu, İbrahim Keklikçioğlu, Derya Poyraz, Selcan Sınacı, Ilgın Türkçüoğlu

**Affiliations:** 1 Gaziantep Şehir Hastanesi, 27470 Gaziantep, Turkey; ibrahimtaskum@gmail.com (İ.T.);; 2 Cengiz Gökçek Kadın Hastalıkları, Doğum ve Çocuk Hastalıkları Hastanesi, 27470 Gaziantep, Turkey

**Keywords:** infertility, sleep quality, psychological resilience, attachment, in vitro fertilization

## Abstract

**Objective:** To compare sleep quality, psychological resilience, and attachment dimensions between women with unexplained infertility and those with male factor infertility undergoing in vitro fertilization (IVF). **Materials and Methods:** In this single-center, cross-sectional study, 201 women undergoing IVF (101 with unexplained and 100 with male factor infertility) completed the Pittsburgh Sleep Quality Index (PSQI), the Brief Resilience Scale (BRS), and the Experiences in Close Relationships–Relationship Structures (ECR-RS) questionnaire. Poor sleep quality was defined as a global PSQI score greater than 5. The primary outcome was the global PSQI score. Between-group comparisons were supplemented by multivariable analyses adjusted for age, economic status, chronic disease, infertility duration, and number of previous IVF attempts. **Results:** No statistically significant differences were observed in most baseline characteristics, although low economic status was more frequent in the male factor group (34.0% vs. 15.8%; *p* = 0.008). The median global PSQI score did not differ significantly between the unexplained and male factor groups (6.0 vs. 5.0; *p* = 0.143). Poor sleep quality was observed in 51.5% and 42.0% of women, respectively (*p* = 0.178), with an overall prevalence of 46.8% (95% CI, 40.0–53.7%). Psychological resilience (3.33 vs. 3.17; *p* = 0.474), attachment avoidance (9.0 vs. 9.5; *p* = 0.336), and attachment anxiety (3.0 vs. 3.0; *p* = 0.648) did not differ significantly between the groups. After adjustment, unexplained infertility was not significantly associated with global PSQI score (β = 0.76, 95% CI −0.17 to 1.69; *p* = 0.110) or poor sleep quality (adjusted prevalence ratio = 1.24, 95% CI 0.92–1.67; *p* = 0.162). **Conclusions:** Women with unexplained and male factor infertility undergoing IVF showed similar patterns of sleep quality, psychological resilience, and attachment dimensions, with no statistically significant between-group differences detected. Poor sleep quality affected nearly half of the study population. These findings should not be interpreted as evidence of equivalence between infertility etiologies but highlight the potential relevance of sleep assessment in women undergoing IVF.

## 1. Introduction

According to the World Health Organization, infertility affects nearly one in six adults globally, corresponding to approximately 17.5% of the adult population worldwide [1]. Infertility is defined as the inability to achieve a clinical pregnancy after 12 months of regular unprotected sexual intercourse [2]. Male factor infertility accounts for approximately 20–30% of infertility cases as a sole cause and contributes to nearly half of all infertility diagnoses worldwide when combined factors are considered [3].

Infertility may arise from male, female, combined, or unexplained factors. Despite advances in diagnostic evaluation, the underlying cause of infertility cannot be identified in a considerable proportion of couples, and these cases are classified as unexplained infertility, which remains one of the most challenging diagnostic categories due to the absence of an identifiable cause. Diagnostic uncertainty may increase emotional distress and psychological burden among affected women.

Infertility is a major health problem affecting millions of couples worldwide and is frequently associated with considerable psychological distress. In addition to its reproductive implications, infertility may negatively influence emotional well-being, interpersonal relationships, and quality of life [4]. Previous studies have demonstrated that infertile women experience higher levels of anxiety, depression, stress, and emotional burden compared with fertile controls [5,6]. Furthermore, the relationship between infertility and psychological distress appears to be bidirectional, as infertility may increase emotional burden, while psychological distress itself may adversely affect reproductive function and treatment outcomes [7,8]. Infertility may impose substantial psychological pressure on women due to social stigma, marital difficulties, and challenges related to self-identity [9]. A recent meta-analysis including more than 124,000 women demonstrated that women with infertility experience approximately 1.6-fold higher levels of psychological distress compared with the general female population [10]. Increased psychological distress in infertile women may negatively influence treatment outcomes, interpersonal and family relationships, and, in severe cases, may even lead to suicidal thoughts or behaviors [8,11,12].

Women experiencing infertility are frequently exposed to substantial infertility-related stress throughout the diagnostic and treatment process. Sources of stress may include disruptions in daily functioning, marital difficulties, concerns regarding intimate relationships, and social pressure from family members and peers [13]. The experience of infertility and the associated diagnostic and therapeutic processes may constitute substantial sources of emotional burden, often challenging an individual’s psychological well-being and coping capacity [14].

Sleep disturbances are increasingly recognized as an important component of the psychological burden associated with infertility. Poor sleep quality has been reported to be highly prevalent among infertile women and may adversely affect emotional regulation, stress perception, and overall well-being [15,16]. Previous studies have reported that approximately one-third of infertile women experience sleep disturbances or poor sleep quality [17]. Furthermore, impaired sleep quality has also been associated with unfavorable assisted reproductive treatment outcomes in some studies. Sleep disturbances may represent a clinically relevant indicator of impaired psychological well-being among infertile women [18]. Despite increasing interest in the relationship between sleep and fertility, the available evidence remains limited, and the underlying mechanisms linking sleep quality to reproductive health have not yet been fully elucidated [19].

Psychological resilience, defined as the ability to adapt successfully to stress and adversity, may play a protective role in infertile individuals. As a positive psychological resource, resilience may facilitate adaptation to infertility-related challenges and promote psychological well-being [20,21]. Psychological resilience refers not only to successful adaptation to adversity but also to the ability to maintain or regain stable psychological functioning in the face of stressful life events [22]. Individuals with higher levels of resilience are generally better able to adapt to stressful life events and recover more effectively from adversity [23]. Higher resilience levels have been associated with improved psychological adjustment, better coping strategies, and enhanced quality of life in infertility populations [21,24]. Previous studies have demonstrated that higher resilience levels are associated with lower psychological distress and fewer sleep-related problems, suggesting a potential protective role against the adverse emotional consequences of infertility [25,26,27]. Additionally, resilience may buffer the negative effects of infertility-related stress on sleep and emotional health [28].

Attachment dimensions are another important psychological factor that may influence emotional responses to infertility. Infertility may evoke feelings of vulnerability, uncertainty, and an increased need for emotional support, thereby activating the attachment system [29]. According to attachment theory, stressful or threatening situations activate the attachment system. Attachment orientations may subsequently shape emotional responses and the strategies used to regulate distress [30,31,32,33]. Adult attachment is commonly conceptualized along two dimensions: attachment anxiety, characterized by fears of rejection and abandonment, and attachment avoidance, characterized by discomfort with emotional intimacy and dependence on others [34]. Previous research suggests that individuals exhibiting insecure attachment orientations are more likely to interpret stressful experiences negatively and may therefore be at greater risk of developing psychological distress compared with securely attached individuals [35,36]. Insecure attachment patterns, particularly anxious attachment, have been associated with increased infertility-related distress, depressive symptoms, and maladaptive coping mechanisms [37,38]. Previous studies have demonstrated that attachment dimensions are associated with infertility-related distress and may influence psychological adjustment among infertile individuals [29,39]. Recent evidence also suggests that attachment dimensions may affect interpersonal adjustment and psychological functioning among infertile couples [40].

Among infertility etiologies, unexplained infertility may present distinct psychological challenges because of uncertainty regarding the underlying cause. The absence of a clearly identifiable medical explanation may influence how women experience and cope with the infertility process. In contrast, women with male factor infertility may experience different psychological responses due to differences in perceived responsibility and attribution of infertility.

Although previous studies have separately investigated sleep quality, psychological resilience, and attachment dimensions in infertility populations, little is known about how these factors coexist in women with different infertility etiologies. In particular, data comparing women with unexplained infertility and male factor infertility remain scarce. Therefore, the present study aimed to compare sleep quality, psychological resilience, and attachment dimensions among women with unexplained infertility and those with male factor infertility. The primary outcome was global sleep quality, assessed using the Pittsburgh Sleep Quality Index (PSQI) total score, while psychological resilience, attachment dimensions, and poor sleep status were evaluated as secondary outcomes.

## 2. Materials and Methods

### 2.1. Study Design and Setting

This single-center, cross-sectional study was conducted at Gaziantep City Hospital, Gaziantep, Türkiye, between March and September 2025. Women who were undergoing in vitro fertilization (IVF) treatment during the study period and who met the eligibility criteria were consecutively invited to participate.

### 2.2. Participants

The final analytic sample comprised 201 women undergoing IVF, including 101 women with unexplained infertility and 100 women with male factor infertility. Women were eligible if they were at least 18 years of age, had a confirmed diagnosis of either unexplained or male factor infertility, had primary infertility with no history of previous pregnancy, were undergoing IVF, were able to read and complete the study questionnaires, and provided written informed consent. Women were excluded if they had a previously diagnosed psychiatric disorder or were currently using psychotropic medication, had a previously diagnosed primary sleep disorder, were currently pregnant, or were engaged in night-shift work. Women with female-factor or combined infertility and those who provided incomplete questionnaire data were also not included. The presence of previously diagnosed psychiatric disorders, primary sleep disorders, and relevant medication use was assessed based on participant medical history and review of available hospital medical records.

Unexplained infertility was defined as the failure to achieve pregnancy after a standard fertility evaluation without an identifiable female or male cause. The evaluation included regular ovulatory menstrual cycles, assessment of ovarian reserve using serum anti-Müllerian hormone (AMH) levels and antral follicle count (AFC), at least one patent fallopian tube documented by hysterosalpingography, no clinically relevant uterine abnormality on transvaginal ultrasonography, and a semen analysis without clinically relevant abnormalities in the male partner [41]. Male factor infertility was defined by abnormalities in sperm concentration, motility, and/or morphology based on semen analysis performed according to the sixth edition (2021) of the World Health Organization Laboratory Manual for the Examination and Processing of Human Semen [42]. Women in the male-factor infertility group had no identified female cause of infertility, and couples with combined female and male infertility factors were excluded.

### 2.3. Ethical Considerations

The study was conducted in accordance with the principles of the Declaration of Helsinki and was approved by the Gaziantep City Hospital Non-Interventional Clinical Research Ethics Committee (decision no. 153/2025; date: 27 February 2025). Written informed consent was obtained from all participants prior to enrollment.

### 2.4. Data Collection and Measures

All questionnaires were completed by the participants at the outpatient clinic at baseline, before the initiation of IVF treatment or ovarian stimulation. The timing of questionnaire administration was the same for all participants.

After providing informed consent, participants completed a sociodemographic and clinical data form, the Pittsburgh Sleep Quality Index, the Brief Resilience Scale, and the Experiences in Close Relationships–Relationship Structures questionnaire. The questionnaires were completed by the participants in the outpatient clinic during their visit.

Sociodemographic and clinical data form. A structured form developed by the researchers was used to record age, education level, employment status, self-reported economic status, duration of infertility, number of previous IVF attempts, history of previous IVF treatment, and the presence of chronic disease.

Pittsburgh Sleep Quality Index (PSQI). Sleep quality over the preceding month was assessed using the PSQI, a 19-item self-report instrument [43]. The items generate seven component scores—subjective sleep quality, sleep latency, sleep duration, habitual sleep efficiency, sleep disturbances, use of sleep medication, and daytime dysfunction—each scored from 0 to 3. The component scores are summed to yield a global score ranging from 0 to 21, with higher scores indicating poorer sleep quality, and a global score greater than 5 was used to define poor sleep quality. The validity and reliability of the Turkish version were established by Ağargün et al. [44].

Brief Resilience Scale (BRS). Psychological resilience was measured using the BRS, a six-item self-report scale that assesses the ability to recover from stress [45]. Items are rated on a 5-point Likert scale; after reverse scoring items 2, 4, and 6, the mean of all six items is computed, with higher scores reflecting greater resilience. The Turkish adaptation was performed by Doğan [46].

Experiences in Close Relationships–Relationship Structures (ECR-RS) questionnaire. Adult attachment orientations were assessed using the ECR-RS [47]. The questionnaire yields two dimensional scores: attachment avoidance, derived from items 1–6 (items 1–4 reverse scored), and attachment anxiety, derived from items 7–9, with higher scores indicating greater insecurity along each dimension. The Turkish validity and reliability study was conducted by Deveci Şirin and Şen Doğan [48]. Participants completed the ECR-RS with reference to their spouse or romantic partner. Attachment avoidance was calculated by summing the six avoidance items after reverse-scoring the first four items, yielding a possible score range of 6–42. Attachment anxiety was calculated by summing the three anxiety items, yielding a possible score range of 3–21. Higher scores indicated higher levels of attachment avoidance or anxiety, respectively.

### 2.5. Sample Size

No formal a priori sample size calculation was performed. We aimed to recruit approximately 100 women in each diagnostic group. To account for potentially incomplete or unusable questionnaires, 102 participants were recruited in each group. Following data-quality assessment, one questionnaire in the unexplained infertility group and two questionnaires in the male-factor infertility group were excluded because of incomplete or unusable responses. The final analytic sample therefore comprised 201 women (101 with unexplained infertility and 100 with male-factor infertility). No item-level missing data were present among the 201 participants included in the final analysis; therefore, no data imputation was required.

A sensitivity analysis based on the available sample size (101 women with unexplained infertility and 100 women with male-factor infertility), a two-sided α of 0.05, and 80% power indicated that the study could detect a standardized between-group effect size of approximately Cohen’s d = 0.40. An equivalence analysis was not performed because a clinically validated equivalence margin for between-group differences in global PSQI scores among women undergoing IVF has not been established. Accordingly, non-significant between-group differences were not interpreted as evidence of equivalence [49].

### 2.6. Statistical Analysis

Statistical analyses were performed using R software (version 4.5.0). The distribution of continuous variables was assessed using the Shapiro–Wilk test and visual inspection of distributional plots. Normally distributed continuous variables were summarized as mean ± standard deviation (SD), whereas non-normally distributed continuous variables were summarized as median and interquartile range (IQR). Categorical variables were presented as number and percentage [*n* (%)]. Between-group comparisons were performed using Welch’s independent-samples *t* test or the Mann–Whitney U test for continuous variables, according to data distribution, and the chi-square test or Fisher’s exact test, as appropriate, for categorical variables.

The global Pittsburgh Sleep Quality Index (PSQI) score was designated as the primary outcome. Poor sleep prevalence (global PSQI > 5), Brief Resilience Scale (BRS) score, attachment avoidance, and attachment anxiety were considered secondary outcomes. The seven individual PSQI component scores were considered exploratory outcomes. To account for multiple comparisons across the seven exploratory PSQI components, *p* values were adjusted using the Holm method.

Effect sizes for unadjusted comparisons of continuous study outcomes were expressed as rank-biserial correlations. For the primary and secondary outcomes, additional regression analyses were performed to account for potential confounding. Models were adjusted for age, economic status, chronic disease, infertility duration, and number of previous IVF attempts. Continuous outcomes (global PSQI, BRS, attachment avoidance, and attachment anxiety) were analyzed using linear regression with HC3 heteroscedasticity-robust standard errors, and results were reported as regression coefficients (β) with 95% confidence intervals (CIs). Poor sleep (PSQI > 5) was analyzed using modified Poisson regression with robust variance, with results reported as prevalence ratios (PRs) and 95% CIs. Male-factor infertility was used as the reference group in all regression models.

Internal consistency in the present sample was evaluated using Cronbach’s alpha for the PSQI, BRS, attachment avoidance, and attachment anxiety measures. All statistical tests were two-sided, and a *p* value < 0.05 was considered statistically significant.

## 3. Results

A total of 201 women undergoing in vitro fertilization (IVF) were included in the study, comprising 101 women with unexplained infertility and 100 women with male factor infertility. The baseline demographic and clinical characteristics of the participants are presented in Table 1. No statistically significant between-group differences were detected in the baseline characteristics, except for self-reported economic status. A higher proportion of women in the male factor infertility group reported low economic status (34.0% vs. 15.8%; *p* = 0.008).

**Figure 1:**
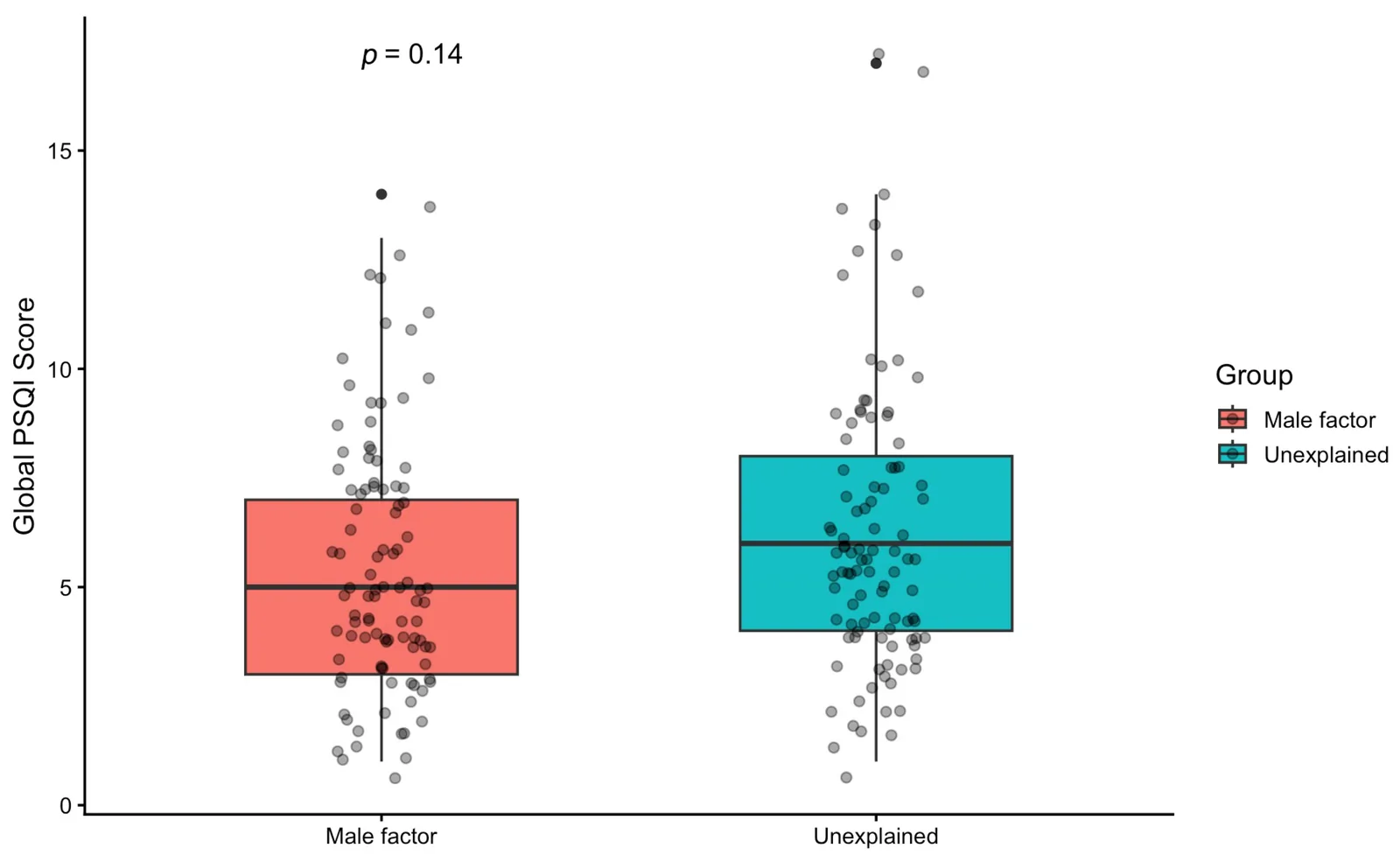
Comparison of global Pittsburgh Sleep Quality Index (PSQI) scores between women with unexplained infertility and those with male factor infertility. Boxes represent the median and interquartile range; individual data points are overlaid. The between-group difference was not statistically significant (Mann–Whitney U test, *p* = 0.14).

Sleep quality, psychological resilience, and attachment dimensions are summarized in Table 2. The median global PSQI score was 6.0 (IQR, 4.0–8.0) in the unexplained infertility group and 5.0 (IQR, 3.0–7.0) in the male factor infertility group (U = 5651.0, *p* = 0.143, rank-biserial r = 0.119) (Figure 1). Poor sleep quality (global PSQI > 5) was identified in 52 (51.5%) women with unexplained infertility and 42 (42.0%) women with male factor infertility (*p* = 0.178). Overall, 94 of 201 participants (46.8%, 95% CI 40.0–53.7%) had poor sleep quality. No statistically significant between-group differences were detected in psychological resilience, attachment avoidance, or attachment anxiety (Table 2).

None of the seven exploratory PSQI component scores differed significantly between the groups, and all comparisons remained non-significant after adjustment for multiple testing using the Holm method (all Holm-adjusted *p* > 0.05; Table 3).

Internal consistency coefficients in the present sample were α = 0.637 for the PSQI, α = 0.590 for the BRS, α = 0.780 for attachment avoidance, and α = 0.845 for attachment anxiety.

In the adjusted analyses, infertility etiology was not significantly associated with any of the primary or secondary outcomes (Table 4). After adjustment for age, economic status, chronic disease, infertility duration, and number of previous IVF attempts, the estimated difference in global PSQI score for women with unexplained infertility relative to those with male-factor infertility was β = 0.76 (95% CI −0.17 to 1.69; *p* = 0.110). The adjusted prevalence ratio for poor sleep was 1.24 (95% CI 0.92–1.67; *p* = 0.162). No statistically significant adjusted associations were observed for psychological resilience, attachment avoidance, or attachment anxiety (Table 4).

## 4. Discussion

In this cross-sectional study of 201 women undergoing IVF, no statistically significant differences were detected between women with unexplained infertility and those with male-factor infertility in global sleep quality, poor sleep prevalence, psychological resilience, or attachment avoidance and anxiety. These findings remained largely unchanged after adjustment for age, economic status, chronic disease, infertility duration, and number of previous IVF attempts. Importantly, poor sleep was common in the overall study population, affecting 46.8% of participants. However, the absence of statistically significant between-group differences should not be interpreted as evidence of equivalence between the two infertility groups.

The prevalence of poor sleep quality observed in our cohort (51.5% in the unexplained and 42.0% in the male factor group) was higher than the approximately one-third prevalence reported in some previous studies [15,17]. This high burden is clinically relevant, as impaired sleep has been associated not only with poorer emotional regulation and stress perception [16] but also with less favorable assisted reproductive treatment outcomes [18]. Consistent with these findings, a prospective cohort study of women assessed before ART treatment reported that adverse sleep characteristics were associated with poorer oocyte, fertilization, and clinical pregnancy outcomes [50]. No statistically significant difference in global PSQI scores was detected between the two etiological groups. Although shared psychological and physiological demands related to infertility and its treatment may contribute to sleep disturbance, the present findings do not establish that sleep disturbance is independent of infertility etiology. Indeed, sleep and reproductive function are interconnected through shared neuroendocrine pathways involving the hypothalamic–pituitary–gonadal and hypothalamic–pituitary–adrenal axes, which may contribute to vulnerability to sleep disruption among women experiencing infertility [19]. In the exploratory analysis of individual PSQI components, no statistically significant between-group differences were detected after correction for multiple comparisons.

Psychological resilience did not differ between the groups, with median Brief Resilience Scale scores of 3.33 in the unexplained infertility group and 3.17 in the male factor infertility group. Resilience has been conceptualized as a positive psychological resource that facilitates adaptation to infertility-related stress and is associated with better quality of life and lower psychological distress [20,21,24]. No statistically significant between-group difference in resilience was detected, although this finding should not be interpreted as evidence of equivalent resilience across infertility etiologies. Previous research has suggested that resilience may buffer the adverse emotional and sleep-related consequences of infertility and may mediate the relationship between sleep quality and psychological distress [28]. However, the present study did not examine associations, mediation, or moderation among resilience, sleep, and attachment; therefore, no conclusions regarding such relationships can be drawn from our findings.

Attachment avoidance and attachment anxiety did not differ significantly between the groups. Insecure attachment patterns have been linked to greater infertility-related distress, depressive symptoms, and maladaptive coping [37,38], and attachment dimensions have been reported to influence psychological adjustment among infertile individuals [39,40]. More recently, a study of women undergoing IVF found that attachment anxiety and avoidance mediated the association between infertility-related stress profiles and psychological distress [51]. In the present study, no statistically significant between-group differences were detected in either attachment dimension; however, this finding does not establish that attachment dimensions are equivalent across infertility etiologies. It is plausible that attachment orientation, as a relatively stable and trait-like characteristic, is shaped by developmental and relational factors in addition to experiences related to infertility [29]. Further studies incorporating broader psychosocial and relational factors, including dyadic assessments of both partners, may help clarify whether infertility etiology is associated with attachment dimensions.

Taken together, the present findings indicate that no statistically significant differences were detected between the two infertility groups in the specific constructs assessed: sleep quality, psychological resilience, and attachment dimensions. Although women with male factor infertility may experience different psychological responses owing to differences in the perceived locus of responsibility, such differences were not evident in the outcomes measured in this study. Previous studies have reported that infertility etiology may show limited associations with mood disturbance and life satisfaction among couples undergoing assisted reproduction [52], while women may experience substantial psychological burden across different infertility contexts [53]. These observations provide a broader context for our findings but do not establish that psychological distress is equivalent or independent of infertility etiology. The diagnostic and treatment process itself may represent an important source of stress for women undergoing IVF [14].

An important consideration in interpreting our findings is the significant between-group difference in self-reported economic status, with a higher proportion of women in the male factor group reporting low economic status. Socioeconomic disadvantage is a recognized correlate of poorer sleep quality and greater psychological distress [54]. We therefore included economic status, together with age, chronic disease, infertility duration, and number of previous IVF attempts, in the adjusted models. The adjusted analyses did not materially alter the pattern of findings. Nevertheless, residual confounding by unmeasured socioeconomic, reproductive, lifestyle, and psychological factors cannot be excluded.

From a clinical perspective, the high prevalence of poor sleep quality in both etiological groups supports consideration of routine sleep assessment during IVF care. The potential value of systematic sleep assessment has also been highlighted in other outpatient settings [55], although evidence from psychiatric populations should be extrapolated cautiously to women undergoing IVF. Brief assessment of psychological well-being may also help identify women who could benefit from additional psychological support. This approach is consistent with ESHRE recommendations emphasizing the integration of routine psychosocial care throughout infertility and medically assisted reproduction services [56]. More broadly, recent psychiatric literature has emphasized individualized assessment of psychological characteristics and multidisciplinary, patient-centered models of care, although the applicability of these approaches to infertility care requires specific evaluation [57,58]. Such interventions have been shown to reduce anxiety and depressive symptoms and improve well-being among women with infertility [59]. However, the present findings do not establish equivalence between infertility etiologies, and larger, adequately powered, multicenter, and dyadic studies with comprehensive adjustment for potential confounders are needed to determine whether clinically meaningful subgroup differences exist.

This study has several strengths, including a relatively large and well-characterized sample of women undergoing IVF, the inclusion of two clearly defined etiological groups, and the simultaneous use of validated instruments assessing sleep quality, psychological resilience, and attachment dimensions. Several limitations should nonetheless be acknowledged. First, the cross-sectional design precludes causal inference. Second, all outcomes were based on self-report questionnaires, and objective measures of sleep were not obtained. In addition, specific measures of depression, anxiety, or psychological distress were not administered; therefore, the findings should not be interpreted as reflecting overall psychological morbidity. Third, this was a single-center study including only women, without assessment of their male partners, limiting generalizability and precluding couple-level analyses. Furthermore, the restrictive eligibility criteria, including the exclusion of women with diagnosed psychiatric disorders, psychotropic medication use, primary sleep disorders, and night-shift work, may have introduced selection bias and may have resulted in an underestimation of the real-world burden of poor sleep among women undergoing IVF. Fourth, although the adjusted analyses accounted for age, economic status, chronic disease, infertility duration, and the number of previous IVF attempts, residual confounding remains possible. In particular, potentially relevant factors including body mass index, smoking, alcohol and caffeine consumption, physical activity, and previous pregnancy loss were not systematically collected and therefore could not be included in the adjusted analyses. Although questionnaires were administered at baseline before IVF treatment, more detailed timing in relation to the menstrual cycle and specific stages of the IVF process was not assessed, which may be relevant because psychological and sleep-related experiences may vary across treatment stages. Fifth, a formal screening log was not maintained; therefore, the number of potentially eligible women who were not enrolled or declined participation was not recorded. Sixth, the individual PSQI component analyses were exploratory; although the corresponding *p* values were adjusted for multiple comparisons using the Holm method, these findings should be interpreted cautiously. Seventh, the internal consistency of some instruments, particularly the BRS, was modest in the present sample, which may have reduced measurement precision. Finally, the absence of a fertile comparison group limits the ability to contextualize the observed levels of sleep disturbance and psychological characteristics.

In conclusion, no statistically significant differences were detected between women with unexplained and male factor infertility undergoing IVF in sleep quality, psychological resilience, or attachment dimensions, and this pattern remained after adjustment for potential confounders. Poor sleep was common in the study sample, affecting nearly half of the participants. These findings should not be interpreted as demonstrating equivalence between infertility etiologies or as indicating that the psychological consequences of infertility are independent of etiology. Rather, the study did not detect differences between the two groups in the specific constructs measured. The high prevalence of poor sleep supports consideration of sleep assessment as part of IVF care. Larger, multicenter, and longitudinal studies—ideally incorporating both partners, broader psychological and lifestyle assessments, and objective sleep measures—are warranted to determine whether clinically meaningful differences between infertility etiologies exist and to clarify the temporal and mechanistic relationships among sleep, resilience, and attachment in infertile women.

## Figures and Tables

**Table 1:** Baseline characteristics of the study groups.

Variable	Unexplained Infertility (n = 101)	Male Factor Infertility (n = 100)	Test Statistic	p Value
Age (years)	29.54 ± 4.53	29.63 ± 4.83	t = −0.129 (df = 197.90)	0.898
Duration of infertility (years)	4.0 (2.0–6.0)	3.0 (2.0–6.0)	U = 5702.0	0.112
Previous IVF attempts	0 (0–1)	0 (0–0)	U = 5328.0	0.384
Education level			χ^2^ = 1.549 (df = 4)	0.818
Primary school	13 (12.9)	11 (11.0)		
Secondary school	12 (11.9)	18 (18.0)		
High school	37 (36.6)	34 (34.0)		
University	34 (33.7)	32 (32.0)		
Postgraduate	5 (5.0)	5 (5.0)		
Employment status			Fisher’s exact test	0.867
Employed	22 (21.8)	23 (23.0)		
Unemployed	79 (78.2)	77 (77.0)		
Economic status			χ^2^ = 9.671 (df = 2)	0.008
Low	16 (15.8)	34 (34.0)		
Middle	78 (77.2)	63 (63.0)		
High	7 (6.9)	3 (3.0)		
Previous IVF treatment			Fisher’s exact test	0.630
Yes	28 (27.7)	24 (24.0)		
No	73 (72.3)	76 (76.0)		
Chronic disease			Fisher’s exact test	0.081
Yes	5 (5.0)	12 (12.0)		
No	96 (95.0)	88 (88.0)		

Data are presented as mean ± standard deviation (SD), median (interquartile range [IQR]), or *n* (%), as appropriate. Continuous variables were compared using Welch’s independent-samples *t* test or the Mann–Whitney U test according to data distribution. Categorical variables were compared using Pearson’s chi-square test or Fisher’s exact test, as appropriate. Degrees of freedom are reported where applicable. A two-sided *p* value < 0.05 was considered statistically significant.

**Table 2:** Comparison of sleep quality, psychological resilience, and attachment dimensions between the study groups.

Variable	Unexplained Infertility (n = 101)	Male Factor Infertility (n = 100)	Test Statistic	p Value
PSQI total score	6 (4–8)	5 (3–7)	U = 5651.0	0.143
Poor sleep (PSQI > 5), *n* (%)	52 (51.5)	42 (42.0)	χ^2^ = 1.816 (df = 1)	0.178
Brief Resilience Scale mean score	3.33 (2.83–3.83)	3.17 (2.83–3.67)	U = 5344.5	0.474
Attachment avoidance score	9 (6–16)	9.5 (6–18)	U = 4664.0	0.336
Attachment anxiety score	3 (3–8)	3 (3–7)	U = 4885.5	0.648

Note: Data are presented as median (interquartile range [IQR]) or *n* (%), as appropriate. Continuous outcomes were compared using the Mann–Whitney U test, and poor sleep prevalence was compared using Pearson’s chi-square test. A two-sided *p* value < 0.05 was considered statistically significant. PSQI, Pittsburgh Sleep Quality Index.

**Table 3:** Comparison of PSQI component scores between groups.

PSQI Component	Unexplained Infertility (n = 101)	Male Factor Infertility (n = 100)	U	p Value	Holm-Adjusted p Value
Subjective sleep quality	1 (1–1)	1 (0–1)	5655.5	0.090	0.540
Sleep latency	1 (1–2)	1 (1–2)	5731.5	0.075	0.525
Sleep duration	0 (0–1)	0 (0–1)	5053.5	0.993	1.000
Habitual sleep efficiency	1 (0–2)	0 (0–2)	5612.5	0.140	0.700
Sleep disturbances	1 (1–2)	1 (1–2)	5228.5	0.613	1.000
Use of sleep medication	0 (0–0)	0 (0–0)	5136.5	0.611	1.000
Daytime dysfunction	1 (0–1)	0 (0–1)	5470.0	0.261	1.000

Note: Data are presented as median (interquartile range [IQR]). Between-group comparisons were performed using the Mann–Whitney U test. *p* values were adjusted for multiple comparisons across the seven PSQI components using the Holm method. A two-sided Holm-adjusted *p* value < 0.05 was considered statistically significant. PSQI, Pittsburgh Sleep Quality Index.

**Table 4:** Unadjusted and adjusted associations of infertility etiology with sleep quality, psychological resilience, and attachment dimensions.

Outcome	Unadjusted Effect Estimate (95% CI)	p Value	Adjusted Effect Estimate (95% CI)	p Value
Global PSQI score	β = 0.72 (−0.14 to 1.58)	0.102	β = 0.76 (−0.17 to 1.69)	0.110
Poor sleep (PSQI > 5)	PR = 1.23 (0.91–1.65)	0.181	aPR = 1.24 (0.92–1.67)	0.162
Brief Resilience Scale score	β = 0.08 (−0.11 to 0.27)	0.394	β = 0.05 (−0.16 to 0.25)	0.646
Attachment avoidance	β = −2.04 (−4.32 to 0.25)	0.080	β = −1.82 (−4.25 to 0.61)	0.142
Attachment anxiety	β = −0.13 (−1.53 to 1.28)	0.858	β = −0.05 (−1.51 to 1.41)	0.945

Note: Male-factor infertility was the reference group; therefore, β coefficients represent the estimated difference for unexplained infertility relative to male-factor infertility. Adjusted models included age, economic status, chronic disease, infertility duration, and number of previous IVF attempts. Continuous outcomes were analyzed using linear regression with HC3 robust standard errors. Poor sleep (PSQI > 5) was analyzed using modified Poisson regression with robust variance and is reported as a prevalence ratio (PR). CI, confidence interval; PSQI, Pittsburgh Sleep Quality Index; PR, prevalence ratio; aPR, adjusted prevalence ratio.

## Data Availability

The datasets generated and analyzed during the current study are available from the corresponding author (F.T.K.) upon reasonable request.
